# Individual Brain Charting, a high-resolution fMRI dataset for cognitive mapping

**DOI:** 10.1038/sdata.2018.105

**Published:** 2018-06-12

**Authors:** Ana Luísa Pinho, Alexis Amadon, Torsten Ruest, Murielle Fabre, Elvis Dohmatob, Isabelle Denghien, Chantal Ginisty, Séverine Becuwe-Desmidt, Séverine Roger, Laurence Laurier, Véronique Joly-Testault, Gaëlle Médiouni-Cloarec, Christine Doublé, Bernadette Martins, Philippe Pinel, Evelyn Eger, Gaël Varoquaux, Christophe Pallier, Stanislas Dehaene, Lucie Hertz-Pannier, Bertrand Thirion

**Affiliations:** 1 Parietal Team, Inria, Saclay, France; 2 Neurospin, CEA, Saclay, France; 3 Paris-Saclay University, Paris, France; 4 Cognitive Neuroimaging Unit, Saclay, France; 5 INSERM, Paris, France; 6 Paris-Sud University, Paris, France; 7 UNIACT-U1129, Paris, France; 8 Collège de France, Paris, France; 9 Paris Descartes University, Paris, France

**Keywords:** Attention, Magnetic resonance imaging, Decision, Language, Cognitive control

## Abstract

*Functional Magnetic Resonance Imaging* (fMRI) has furthered brain mapping on perceptual, motor, as well as higher-level cognitive functions. However, to date, no data collection has systematically addressed the functional mapping of cognitive mechanisms at a fine spatial scale. The *Individual Brain Charting* (IBC) project stands for a high-resolution multi-task fMRI dataset that intends to provide the objective basis toward a comprehensive functional atlas of the human brain. The data refer to a cohort of 12 participants performing many different tasks. The large amount of task-fMRI data on the same subjects yields a precise mapping of the underlying functions, free from both inter-subject and inter-site variability. The present article gives a detailed description of the first release of the IBC dataset. It comprises a dozen of tasks, addressing both low- and high- level cognitive functions. This openly available dataset is thus intended to become a reference for cognitive brain mapping.

## Background & Summary

Mapping functional neuroanatomy has become a central challenge in cognitive neuroscience and it constitutes an essential step toward linking brain systems and behavior. Neuroimaging techniques, like *Functional Magnetic Resonance Imaging* (fMRI), have contributed to the investigation of brain regions involved in a variety of cognitive processes.

Establishing an atlas of brain functions calls for probing many different cognitive tasks. Yet, such coverage has only been possible by compounding different studies, like in meta-analyses. This approach is however susceptible to many sources of variability, such as between-protocol and inter-subject variability. The latter problem is common to all group-level analyses. It has been shown to undermine the estimation of statistical significance^1,2^ and the ascription of brain locations to specific functions^3,4^. A handful of studies in neuroimaging has adopted individual analysis, in order to overcome both functional and anatomical inter-subject variability^3–9^.

To avoid sources of inter-subject plus inter-site variability across tasks, the *Individual Brain Charting* (IBC) project aims at providing an heterogeneous task-fMRI dataset acquired in a fixed environment. The data are obtained from a permanent cohort of 12 participants during the performance of a dozen of tasks. Given its task-wise organization, the IBC dataset encompasses a wide range of psychological domains that will yield a refined characterization of the neurocognitive mechanisms underlying human behavior. Raw and post-processed data from within-subject level analysis are made available along with task-stimuli. The data are acquired at high-spatial resolution (1.5mm for fMRI data) to enable accurate delineations of brain regions and elicit finer macroscopic representations of functional networks.

There has been a strong uptake in large-scale brain imaging datasets. Nonetheless, existing ones differ significantly from the IBC dataset. Many of these collections are centered on resting-state fMRI data^10–14^, including large-scale databases devoted to research in clinical neuroscience^15,16^. Further, the *Human Connectome Project* (HCP) accounts for a considerable amount of subjects scanned with different neuroimaging modalities^17^, including not only resting-state but also task-fMRI data. This component of the project sought to delineate and characterize representative functional territories, according to their implication in task performance on a large population size^18,19^. It comprises however a limited amount of fMRI tasks. The brain atlas was mostly developed from resting-state fMRI data^19^ and emphasis was given to population aspects, like the investigation of between-subject variability with respect to behavior^20^ or genetics^21^. Another example is the *studyforrest* initiative, which provides a multi-modal brain-imaging dataset comprising task-fMRI data on the continuous presentation of audio descriptions from the “Forrest Gump” movie. It has given rise to many studies investigating the neurocognitive encoding of complex auditory information, like the ability to perceive language, music or social interplay^6,22–24^. The tasks employed across these studies were nevertheless restricted to naturalistic stimuli, that can not be easily integrated in more classical controlled experiments. Finally, a forerunner of the IBC project^25^ relates to the collection of a functional localizer task on 81 participants. This task, named *ARCHI Standard*, is used in this first release of the IBC dataset. Part of the data are openly available in the Brainomics/Localizer database^26^. However, this study was dedicated to between-subject comparison^27,28^, rather than fine cognitive mapping, which requires greater amount of tasks.

Here, we provide a description of the first release of the IBC dataset, comprising 59 conditions on several cognitive domains, such as somatotopy, calculation, language comprehension and social cognition. Thus, it accounts for 59 independent contrast maps per participant.

To achieve a comprehensive collection of task-fMRI data, acquisitions are continuously ongoing and more releases are expected over the next years. Although every participant is subject to many *Magnetic Resonance Imaging* (MRI) sessions in the course of approximately seven years, the project does not stand for a longitudinal study. Repeated measurements of the tasks will not be systematically undertaken.

Lastly, the IBC dataset is a free and open dataset devoted to providing a comprehensive characterization of brain systems within the scope of cognitive neuroscience. It is intended to become a reference for human neuroscience and to clarify the relationships between function and structure.

## Methods

To avoid ambiguity with MRI-related terms used throughout this manuscript, definitions of such terms follow the *Brain-Imaging-Data-Structure* (BIDS) Specification version 1.0.0 (ref. 29).

### Participants

The IBC dataset consists of brain MRI data (mostly fMRI) from twelve individuals (two females), ranging in age between 26 and 40 years old (median = 34.5 years) upon recruitment. Handedness was determined with the Edinburgh Handedness Inventory^30^. The corresponding handedness profile of the group is given in Table 1, together with age and sex. The twelve participants were recruited by poster advertisements in the local area. Exclusion criteria were: (i) IQ<80 or IQ>130; (ii) the use of drugs prior to the first exam; (iii) participation in other research protocol involving drugs; (iv) psychiatric and neurologic disorders requiring medication with potential impact on general cognitive abilities; (v) hearing problems; and (vi) any standard MRI counter-indications (for more information on the procedures undertaken concerning the assessment of inclusion criteria, consult section experimental procedure). The experimental procedures were approved by a regional ethical committee for medical protocols in Île-de-France (“Comité de Protection des Personnes” - no. 14-031) and a committee to ensure compliance with data-protection rules (“Commission Nationale de l'Informatique et des Libertés” - DR-2016-033). They were undertaken with the informed written consent of each participant according to the Helsinki declaration and the French public health regulation. Participants were reimbursed on the basis of 80 per MRI acquisition with extra-fees for any additional session.

### Materials

#### Stimulation

For all tasks (see Section experimental paradigms for details), the stimuli were delivered through custom-made scripts that ensure a fully automated environment and computer-controlled collection of the behavioral data. Two software tools were used for the development of such protocols: (1) E-Prime 2.0 Professional (Psychological Software Tools, Inc.); and (2) Expyriment (version 0.7.0, Python 2.7). The visual and auditory stimuli presented in the protocols obtained from the HCP consortium^18^ were translated to french. The auditory material for the *Language processing* task of HCP (see Section experimental paradigms, *HCP Language task* of ref. 18) was synthesized using the Acapela Text To Speech software (Acapela Group). The corresponding material is publicly available, as described in Section code availability.

#### MRI Equipment

The fMRI data were acquired using an MRI scanner Siemens 3T Magnetom Prismafit along with a Siemens Head/Neck 64-channel coil. The *Screening* session (see Table 2 for definition) was performed on a Siemens 3T Magnetom Trio with a 32-channel coil for two participants.

In order to register the behavioral responses from participants during the scanning sessions, two MR-compatible, optic-fiber response devices were interchangeably used according to the type of task employed: (1) a five-button ergonomic pad (Current Designs, Package 932 with Pyka HHSC-1×5-N4); and (2) a pair of in-house custom-made sticks featuring one-top button. MR-Confon package was utilized as audio system in the MRI environment.

All sessions were conducted at the NeuroSpin platform of the CEA Research Institute, Saclay, France.

### Experimental Procedure

Since the IBC initiative stands for a long-term project involving a fixed set of participants, they were subject to a complete assessment before their inclusion in the cohort.

The MRI sessions dedicated to the collection of the present dataset were preceded by three sessions of evaluation. The first two sessions were dedicated to general medical assessment of the participants. They include a checkup following all inclusion criteria as well as additional exams, such as blood sampling, auditory exams and questionnaires.

Blood samples were taken from the subjects during these sessions and, regularly, afterwards at ten time points throughout the study. This procedure is intended to look over potential long-term effects due to repeated MRI acquisitions. An inquiry to assess for prior and/or current consumption of psychotropic substances was performed at the first session; the tetrahydrocannabinol (THC) urine-testing procedure has been employed afterwards, concretely in the beginning of every session, to detect for cannabis usage during the last three days preceding the session. Wechsler Adult Intelligence Scale-III (WAIS-III) tests^31^ were applied during the first session, in order to determine the IQ of the candidate. Furthermore, the Mini DSM-IV interview^32^ was undertaken at the first session, in order to check for psychiatric diseases; another interview takes place midway through the period of the project. Participants were also subjected to auditory tests for verification of sufficiently auditory acuity. The examination was performed by using a diagnostic audiometer (Harp Inventis). These tests have been taken since the first session, at every ten sessions over the course of the project. In addition, the subject filled in the Holmes and Rahe questionnaire^33^ at the first session plus every session that has occurred longer than one month after the previous one; it has been used to detect for personal issues that might compromise the performance of the tasks.

The third session, i.e. the *Screening* session, was subsequently dedicated to a neurological assessment --i.e. detection of potential neurological issues and brain abnormalities-- and behavioral inspection --i.e. small motion amplitude and compliance with given instructions-- as well as the quality check of the MRI data collected.

All remaining sessions pertained to MRI acquisitions entirely devoted to data collection (see Table 2 for details on the organization of the MRI sessions).

The participants first learned about the execution plus timing of the behavioral tasks related to the ongoing MRI session. This short training period took place in a dedicated room outside of the scanner environment. During practice, the participants were seated in a chair in front of a laptop placed on a desk. While the laptop displayed the stimuli following a predetermined sequential order, the participants complied with the corresponding task-related instructions. The procedure and stimuli employed during this training period were identical to the ones used in the MRI session. The training lasted no longer than thirty minutes. Exceptional difficulties concerning execution of the paradigm were neither observed by the experimenters nor reported by the participants. The participants were always scanned in the supine position. Thorough instructions were given to limit head movements and foams were carefully placed within the coil to immobilize the head. With regard to tasks requiring motor responses, the participants used either a pair of one-top button sticks, operated respectively by each hand, or a five-button response box handled uniquely by the right hand (see Section MRI equipment for details). An attachable head-coil double mirror was assembled for presentation of the visual stimuli. In addition, ear plugs were used to reduce scanner noise and headphones were used to allow for verbal communication with the experimenters.

Behavioral tasks were carried out across several runs within the same session (to learn more about the paradigms of the tasks, consult Section experimental paradigms). Each task-related run was repeated in multiples of two, alternating the phase-encoding direction (see Section Imaging Data for details). Each session was dedicated to one or more tasks. The overall temporal structure of the sessions according to the MRI modality employed is detailed in Table 2; specifications about imaging parameters of the referred modalities are described in Section Imaging Data.

The acquisitions were separated by short pauses, ranging between one and three minutes. During these technical breaks, the well-being of the participants was verified through informal interactions and visual inspection of the newly-collected MRI data was carried out for quality-checking.

### Experimental Paradigms

Tasks were aggregated in different sessions according to either already-existing paradigms developed and validated in previous projects^18,25^. The experimental paradigms, i.e. the temporal organization of the tasks, followed a classical event-related design; sometimes, events of the same type were organized in a short sequence, in which case they were formally handled as a block. A few protocols relied on long-lasting events, e.g. the protocols taken from the *HCP Language* or *HCP Social* tasks. Blocks were composed by trials, i.e. cycle of stimuli, typically separated by the display of a fixation cross. All trials within each task were pseudo-randomized in order to avoid the extensively consecutive repetition of trials containing conditions of the same kind.

The following sections are dedicated to a full description of the set of paradigms employed in the study. Note that the material used for stimulus presentation has been made publicly available (see Section code availability), together with video annotations of the corresponding protocols. For each subject, the onset files describing the actual occurrence of events are part of the dataset, following BIDS Specification.

#### *ARCHI* tasks

The *ARCHI* tasks were developed at NeuroSpin in the framework of various neuroimaging projects. Hence, they have been extensively tested and validated by fMRI studies^25,26,34,35^. Data from each task were acquired in two runs, within the same session and using different phase-encoding directions (consult Section Imaging Data and Table 2 for details).

##### ARCHI Standard task

The *ARCHI Standard* task included a variety of elementary cognitive functions, ranging from perceptual processing to high-cognition. The task was organized as a fast event-related paradigm, composed of trials of ten different conditions: (1) left-hand three-times button press, indicated by visual instruction; (2) right-hand three-times button press, indicated by visual instruction; (3) left-hand three-times button press, indicated by auditory instruction; (4) right-hand three-times button press, indicated by auditory instruction; (5) listen to narrative sentences; (6) read narrative sentences; (7) viewing of flashing horizontal checkerboards; (8) viewing of flashing vertical checkerboards; (9) silent subtraction, indicated by visual instruction; and (10) silent subtraction, indicated by auditory instruction.

The task comprised eighty trials in one single run. There were two runs within the same session, in which two different sequences of trials were presented. The sequence of trials per run was pseudo-randomized for the session, but fixed for all participants. The duration of the trials ranged between two and four seconds. There were twenty two epochs of rest between trials, in which a fixation cross was displayed. A complete description of the paradigm is available in ref. 25.

##### ARCHI Spatial

The *ARCHI Spatial* task examined functions involved in spatial cognition. The paradigm was structured in blocks and it comprised five block categories. Each block was formed by a set of trials containing an event, in which visual instructions related to one or two conditions of the same kind were displayed. These five categories of blocks were characterized as follows: (1) saccade, in which ocular movements were performed according to the displacement of a fixation cross from the center toward peripheral locations in the image displayed; (2) imitation of object grasping with the right hand, in which the corresponding object was displayed on the screen; (3) mimic orientation of rhombus, displayed as image background on the screen, using the right hand; events of block categories *2* and *3* featured the same visual stimuli, in order to capture grasping-specific activity; (4) judgment on the left/right orientation of a hand displayed as visual stimulus; and (5) judgment on the palmar/dorsal direction of a hand displayed as visual stimulus. Events of block categories *4* and *5* featured the same visual stimuli.

The task was constituted by forty blocks per run. The order of blocks presentation was pseudo-randomized for the session, but fixed for all participants. Each block was composed by either three or four trials. The duration of the trials ranged between 1.2 and 1.8 s. All blocks were inter-spaced by a fixation-cross period with a duration between four and six seconds.

##### ARCHI Social

The *ARCHI Social* task tackled cognitive functions implicated in social cognition, namely mental abilities linked to the *theory-of-mind* or social interplay. The paradigm was designed in blocks. The blocks were in turn constituted by a set of trials, each of them containing one event. There were eight types of events, that can be described as follows: (1) watch short movies of triangles, exhibiting a putative social interaction; (2) watch short movies of triangles, displaying random movements; (3-4) interpret silently short stories, featuring a *false-belief* plot, which were presented as visual (3) or auditory (4) stimuli; (5-6) interpret silently short stories, featuring a cause-consequence mechanistic plot, which were presented as visual (5) or auditory (6) stimuli; (7) listen passively to short samples of human voices; and *(8)* listen passively to short samples of natural sounds.

The task was constituted by fifteen blocks per run. Each block included one to eight trials. Trials' presentation within a block was pseudo-randomized for the session, but fixed for all participants. The duration of the trials ranged between six and eight seconds. A fixation cross was presented between each block between three and six seconds.

##### ARCHI Emotional

The *ARCHI Emotional* task intended to investigate cognitive-emotional processes on perception of faces and expressions. The paradigm was arranged in blocks that comprised sets of trials. Two categories of conditions can be highlighted from the paradigm: (1) the face-task category and (2) the eye-task category. Both categories involved self-reply according to the stimulus presented at every condition. The face-task category consisted in the presentation of human faces, whereas the eye-task category was dedicated to images representing human eyes. For the face-task category, two conditions corresponded to evaluation of gender and trustworthiness of the human faces. In the eye-task category, the two homologous conditions referred to gender and emotion-expression evaluation. The task concerning emotion expression in the latter category was in accordance with a cue given immediately before the image display. Besides, there was also a baseline condition, common to both categories, showing a gray-scale grid image that may be tilted or not. To achieve the intended goal of this condition, a mental assessment about the orientation of the image had to be performed.

The task consisted of twelve blocks per run, and every block comprised between two and four trials. The order of trials presentation was pseudo-randomized for the session, but fixed for all participants. The trials lasted about 6.3 s. A fixation-cross period of approximately one second was presented between blocks.

#### *HCP* tasks

The *HCP* tasks used herein were reproductions of the task-fMRI paradigms originally developed by ref. 18 for HCP, but with minor changes. The adjustments mainly concerned the translation of all stimuli plus instructions into French, the increment of the number of blocks, which was doubled in most of the tasks (see for each task). In the *HCP Relational* task, the response time per trial was increased after feedback from behavioral pilots. Neither conceptual modifications on the conditions nor other alterations in their temporal sequence were undertaken.

Data from each task were acquired in two runs, within the same session and using different phase-encoding directions (consult Section Imaging Data and Table 2 for details). It follows a short description of the conditions integrating each task over the next sections.

##### HCP Emotion task

The main purpose of the *HCP Emotion* task was to capture neural activity related to the perception of fear and anger. Affective facial expressions were used as visual stimuli due to their importance in adaptive social behavior^36^.

The paradigm included two categories of blocks, namely face and shape blocks. All blocks consisted of a series of events, in which images of faces or shapes were displayed, respectively. There were always three faces/shapes per image; one face/shape was shown at the top and two faces/shapes were shown at the bottom. The participants were then asked to decide which face/shape at the bottom, i.e. left or right face/shape, matched the one displayed at the top, by pressing the corresponding button of the response box.

The task was formed by twelve blocks per run, i.e. six face blocks and six shape blocks. The two block categories were alternately presented for each run. All blocks contained six trials and they were always initiated by a cue of three seconds. In turn, the trials included a visual-stimulus period of two seconds and a fixation-cross period of one second; the total duration of the trial was thus three seconds.

##### HCP Gambling task

This task was adapted from the *Incentive processing* task-fMRI paradigm of the HCP and its aim was to localize brain structures of the reward system, namely the basal ganglia complex.

The paradigm included eight blocks and each block was composed of eight events. For every event, the participants were asked to play a game. The goal was to guess whether the next number to be displayed, which ranged from one to nine, would be smaller or larger than five while a question mark was shown on the screen. The answer was given by pressing the respective button of the response box. Feedback on the correct number was provided afterwards. There was an equal amount of blocks; the participants experienced a majority of either *reward* or *loss* events in each of them.

The task was constituted by eight blocks per run, in which each half related to reward and loss experience, respectively. The order of the two block categories was pseudo-randomized during a single run, but fixed for all participants. A fixation-cross period of fifteen seconds was displayed between blocks. All blocks contained eight trials. The trials included a question-mark visual stimulus lasting up to 1.5 s, a feedback period of one second and a fixation-cross period of one second, as well; the total duration of the trial was then 3.5 s approximately.

##### HCP Motor task

The *HCP Motor* task was designed with the intent of extracting maps on gross motor topography, in particular responses associated with movements of the foot, hand and tongue.

There were thus five categories of motor tasks blocks involving the left foot, the right foot, the left hand, the right hand, and the tongue, respectively. The blocks always started with visual cues referring to which part of the body should be moved. The cues were then followed by a set of events that were indicated by flashing arrows on the screen. The subjects had to move in synchrony with the flashes.

The task was formed by five blocks per category, with a total of twenty blocks per run. The order of the block categories was pseudo-randomized during each run, but fixed for all participants. A fixation-dot period of fifteen seconds was inserted between some blocks. All blocks contained ten trials. Every trial included a cue of one second and a period of performance of twelve seconds.

##### HCP Language task

The *HCP Language* task was used as a localizer of brain regions involved in semantic processing. It was adapted from a study dedicated to exploring the particular role of the anterior temporal lobe on semantic integration^37^.

The paradigm comprised two categories of blocks: (1) story blocks and (2) math blocks. Math blocks served as a control condition in this context, since it is likely to involve both auditory processing and attentional demands. Both type of blocks exhibited auditory stimuli in short epochs, which in turn finished with a final question followed by two possible answers. During story blocks, in which participants were presented with Aesop's fables, the final question targeted the topic of the story. Conversely, math blocks showed arithmetic problems for which the correct solution must be selected. The response was provided after the two possible options were displayed, through pressing the corresponding button of the response box. The difficulty levels of the problems, presented for both categories, were adjusted throughout the experiment, in order to keep the participants engaged in the task while ensuring accurate performances^37^.

The task was composed by eleven blocks per run. For the first run, six story blocks and five math blocks were interleaved, respectively. The reverse amount and order of blocks were used during the second run. The number of trials per block varied between one and four. Nevertheless, it was assured that both block categories matched their length of presentation at every run. There was a cue during two seconds at the beginning of each block, indicating its category. The duration of the trials within a block varied from ten to thirty seconds. Finally, the presentation of the auditory stimuli was always accompanied by the display of a fixation cross on the screen throughout the entire run.

##### HCP Relational task

The *HCP Relational* task employed a relational match-to-sample paradigm, featuring a second-order comparison of relations between two pairs of objects. It served primarily as a localizer of the rostrolateral prefrontal cortex, since *relational matching* mechanisms have been shown to yield activity in this region^38^.

Similar to some previous tasks, the paradigm included two categories of blocks, namely *relational-processing* and *control-matching* blocks. All blocks were constituted by a set of events. In the relational-processing block, visual stimuli consisted of images representing two pairs of objects, in which one pair was placed at the top and the other one at the bottom of the image, respectively. Objects within a pair may differ in two features: shape and texture. The participants had to identify whether the pair of objects from the top differed in a specific feature and, subsequently, they were asked to determine whether the pair from the bottom changed along the same feature. For the control block, one pair of objects was displayed at the top of the image and a single object at the bottom of the same image. In addition, a cue was shown in the middle of that image, indicating which feature is relevant. The participants had thus to indicate whether the object from the bottom was matching either of the two objects from the top, according to the feature specified as a cue.

This task included twelve blocks per run, with six blocks per category. Block categories were, in turn, interleaved for display within a run. A fixation-cross period of sixteen seconds was inserted between some blocks. All blocks contained six trials and they were always initiated by a cue of two seconds. The trials were described by a visual-stimulus plus response period followed by a fixation-cross period, lasting up to ten seconds. The duration of the former was nine seconds and 7.6 s during the relational-processing block and control-matching block, respectively.

##### HCP Social task

The *HCP Social* task intended to provide evidence for brain activity related to social cognition.

The paradigm included two categories of blocks, in which movies were presented during short epochs. The movies consisted in triangle-shape clip art, moving in a predetermined fashion. Putative social interactions could be inferred from movements in the so-called *social* condition. In contrast, objects appeared to be randomly moving in the *random* condition.

The task was constituted by ten blocks per run, five for each category, whose order was pseudo-randomized for every run, but fixed for all participants. There was only one trial per block. It consisted of a twenty-second period of video-clip presentation plus three seconds maximum of a response period, indicated by a momentary instruction on the screen. Thus, the total duration of a block was approximately twenty three seconds. A fixation-cross period of fifteen seconds was always displayed between blocks.

##### HCP Working Memory task

The *HCP Working Memory* (HCP WM) task was adapted from the classical *n-back* task to serve as functional localizer for evaluation of structures involved in *working memory* (WM).

The paradigm included two categories of blocks, namely the “0-back” and “2-back” WM-task blocks. They were both equally presented within a run. A cue was always displayed at the beginning of each block, indicating its block type. Blocks were formed by sets of events, during which pictures of faces, places, tools or body parts were shown on the screen. One block was always dedicated to one specific category of pictures and the four categories were always presented during every run.

The task was constituted by sixteen blocks per run, eight per n-back category. Besides, there were four pairs of blocks per visual category. The order of the blocks, regardless of their category and corresponding class of pictures, was pseudo-randomized for every run, but fixed for all participants. A fixation-cross period of fifteen seconds was introduced between some blocks. All blocks contained ten trials; they were initiated by a cue during 2.5 s. Trials included in turn the presentation of a picture for two seconds and a very short fixation-cross period for half of a second; the total duration of one trial was thus 2.5 s.

#### RSVP Language task

To localize the areas implicated in language comprehension, participants were presented with stimuli consisting of sequences of words, pseudowords or non-words. For some conditions, these sequences were composed by well-formed sentences^39,40^. They were presented as visual stimuli, using a *Rapid-Serial-Visual-Presentation* (RSVP) paradigm.

Concretely, there were six experimental conditions featuring the different types of stimuli: i) complex meaningful sentences, containing at least two clauses (e.g. a main and a relative clause); ii) simple meaningful sentences, with only one main clause; iii) *jabberwocky*, obtained from well-formed sentences whose content words were replaced by meaningless, yet pronounceable pseudowords; iv) lists of words; v) lists of pseudowords; and vi) list of non-words (*aka* consonant-strings).

Following each sequence after a short delay, a probe (which could be a word, pseudoword or non-word) was displayed with a 50% probability of having been presented in the sequence. Participants had then to indicate, by pressing one of the two possible response buttons, whether the probe had appeared in the sentence.

One session of data collection comprised six runs; each of them included sixty trials related to the six experimental conditions, i.e. ten trials corresponded to one condition. The order of the trials was pseudo-randomized within and between runs across participants, such that the same experimental condition did not occur in two immediately successive trials.

A trial lasted ten seconds. It started with the display of a fixation cross for two seconds, followed by the display of a blank screen for 0.5 s. Afterwards, a sequence of ten stimuli was presented at a rate of one stimulus per 0.4 s. Further, a blank screen was displayed during a randomly varying period of time between one and 1.5 s, followed by the display of a fixation cross for 0.5 s plus a probe stimulus for 0.5 s. Finally, the stimuli were cleared and a response-time window opened for 2 s.

### Data Acquisition

#### Behavioral Data

To carry out the ARCHI Standard, HCP Emotion, HCP Gambling, HCP Language, HCP Relational, HCP Social, HCP Working Memory and RSVP Language, active responses were required from the participants. The registry of all behavioral data, such as the qualitative responses to different conditions and corresponding response times, was held in log files generated by the stimulus-delivery software. These data are available (see Section Code Availability).

#### Imaging Data

The parameters for all different types of images acquired are given in Table 3. Specifically, a T1-weighted *Magnetization-Prepared-Rapid-Gradient-Echo* (MPRAGE) anatomical image, a T2-weighted image, a T2 *Fluid-Attenuated-Inversion-Recovery* (FLAIR) image, a diffusion-weighted image sequence and between four to six fMRI sequences were acquired during the Screening acquisition (see Table 2). One must notice that the diffusion-weighted images acquired in this session are not intended to be used for tractography.

Functional MRI data were collected using a *Gradient-Echo* (GE) pulse, whole-brain *Multi-Band* (MB) accelerated^41,42^ *Echo-Planar Imaging* (EPI) T2*-weighted sequence with *Blood-Oxygenation-Level-Dependent* (BOLD) contrasts. Two different acquisitions for the same task were always performed using two opposite phase-encoding directions: one from *Posterior to Anterior* (PA) and the other from *Anterior to Posterior* (AP). The main purpose was to ensure within-subject replication of the same tasks, while mitigating potential limitations concerning the distortion-correction procedure.

*Spin-Echo* (SE) EPI-2D image volumes were acquired in order to correct for spatial distortions. Similarly to the GE-EPI sequences, two different acquisitions were also performed using PA and AP phase-encoding direction. Two pairs of SE-EPI PA/AP sequences were always run before and after the GE-EPI sequences (see Table 2). Four image volumes, i.e. one volume per acquisition, were thus collected at every session.

### Data Analysis

#### Image conversion

The acquired DICOM images were converted to NIfTI format using the dcm2nii tool, which can be found at https://www.nitrc.org/projects/dcm2nii. During conversion to NIfTI, all images were fully anonymized: pseudonyms were removed and images were defaced using the mri_deface command line, from the Freesurfer-6.0.0 library.

### Preprocessing

Source data were preprocessed using *PyPreprocess*. This library offers a collection of Python tools to facilitate pipeline runs, reporting and quality check (https://github.com/neurospin/pypreprocess). It is built upon the *Nipype* library^43^ v0.12.1, that in turn launched various commands used to process neuroimaging data. These commands were taken from the *SPM12* software package (Wellcome Department of Imaging Neuroscience, London, UK) v6685, and the *FSL* library (Analysis Group, FMRIB, Oxford, UK) v5.0.

All fMRI images, i.e. GE-EPI volumes, were collected twice with reversed phase-encoding directions, resulting in pairs of images with distortions going in opposite directions (see Section Imaging Data and Table 2 for details). Susceptibility-induced off-resonance field was estimated from the two Spin-Echo EPI volumes in reversed phase-encoding directions. The images were corrected based on the estimated deformation model, using the *topup* tool^44^ implemented in FSL^45^.

Further, the GE-EPI volumes were aligned to each other within each participant. A rigid body transformation was employed, in which the average volume of all images was used as reference^46^. The mean EPI volume was also co-registered onto the corresponding T1-weighted MPRAGE (anatomical) volume for every participant^47^. The individual anatomical volumes were then segmented into tissue types to finally allow for the normalization of both anatomical and functional data^48^. Concretely, the segmented volumes were used to compute the deformation field for normalization to the standard MNI152 space. The deformation field was then applied to the EPI data. In the end, all volumes were resampled to their original resolution, i.e. 1 mm isotropic for the T1-weighted MPRAGE images and 1.5 mm for the EPI images. These images are used in the validation presented in section Data quality.

#### fMRI Model Specification

The fMRI data were analyzed using the *General Linear Model* (GLM). Regressors of the model were designed to capture variations in BOLD response strictly following stimulus timing specifications. They were estimated through the convolution of temporal representations referring to the task-conditions with the canonical *Hemodynamic Response Function* (HRF), defined according to refs 49,50.

The temporal profile of the conditions was characterized by boxcar functions. To build such models, paradigm descriptors grouped in triplets (i.e. onset time, duration and trial type according to BIDS Specification) were determined from the log files' registries generated by the stimulus-delivery software.

To account for small fluctuations in the latency of the HRF peak response, additional regressors were computed based on the convolution of the same task-conditions profile with the time derivative of the HRF.

Nuisance regressors were also added to the design matrix in order to minimize the final residual error. To remove signal variance associated with spurious effects arising from movements, six temporal regressors were defined for the motion parameters. Further, the first five principal components of the signal, extracted from voxels showing the 5% highest variance, were also regressed to capture physiological noise^51^.

In addition, a discrete-cosine transform set was applied for high-pass filtering (cutoff=128 s). Model specification was implemented using *Nistats* library v0.1, a Python module devoted to statistical analysis of fMRI data (https://nistats.github.io), which leverages *Nilearn*^52^, a Python library for statistical learning on neuroimaging data (https://nilearn.github.io/).

#### Model Estimation

In order to restrict GLM parameters estimation to voxels inside functional brain regions, a brain mask was extracted from the mean EPI volume. The procedure implemented in the Nilearn software simply thresholds the mean fMRI image of each subject in order to separate brain tissue from background, and performs then a morphological opening of the resulting image to remove spurious voxels.

Regarding noise modeling, a first-order autoregressive model was used in the maximum likelihood estimation procedure.

A mass-univariate GLM fit was applied separately to the preprocessed GE-EPI data of each run with respect to a specific task. Parameter estimates pertaining to the experimental conditions were thus computed, along with the respective covariance at every voxel. Various contrasts (linear combinations of the effects), were then defined, referring only to differences in evoked responses between either (i) two conditions-of-interest or (ii) one condition-of-interest and baseline. GLM estimation and subsequent statistical analyses were also implemented using Nistats v0.1. fMRI data analysis was first run on unsmoothed data and, afterwards, on data smoothed with a 5mm full-width-at-half-maximum kernel. Such procedure allows for increased *Signal-to-Noise Ratio* (SNR) and it facilitates between-image comparison. The images used in section Relevance of the IBC dataset for brain mapping are based on the smoothed data.

#### Summary Statistics

Because data from each task were collected, per subject, at least in two acquisitions with opposite phase-encoding directions (see Section Imaging Data for details), statistics of their joint effects were calculated in all aforementioned contrasts with as *Fixed-Effects* (FFX) model. Specifically, *t*-tests were computed at every voxel for each contrast, in order to assess the statistical significance of the differences among evoked responses.

For further analyses (see Sections Relevance of the IBC dataset for brain mapping and Brain Coverage of Functional Activity), all images are confined to an average mask of the grey matter across subjects, keeping all voxels with more than 25% average grey matter density across subjects, as obtained from the SPM12 anatomical segmentation. This masking procedure yields 372 k voxels at the chosen resolution.

### Code Availability

Metadata, concerning the stimuli presented during the BOLD fMRI runs, were made available publicly at https://github.com/hbp-brain-charting/public_protocols. They include: (1) the task-stimuli protocols; (2) demo presentations of the tasks as video annotations; (3) instructions to the participants; and (4) scripts to extract paradigm descriptors from log files for the GLM estimation.

The scripts used for data analysis were also made available through Github under the Simplified BSD license: https://github.com/hbp-brain-charting/public_analysis_code.

## Data Records

Both source (raw) MRI data as well as derived statistical maps are openly available (Data Citation 1).

### Source data

Raw data (*aka* source data according to BIDS Specification) of the present release can be accessed from the *OpenfMRI* public repository^53^ under the data accession number *ds*000244 (https://openfmri.org/dataset/ds000244/; see Data Citation 1 for details). This release counts in total for 306GB of MRI data. The corresponding NIfTI files as well as paradigm descriptors and imaging parameters were organized per run for each session following BIDS Specification:

- The data repository is organized in twelve main directories sub-01 to sub-14. Note that sub-03 and sub-10 are not part of the data (see Table 1);
- Data from each subject are then numbered on a per-session basis, following the chronological order of the corresponding acquisitions. Note that this order is not the same for all subjects;
- Acquisitions are organized within session by modality: func, fmap are part of all sessions, whereas anat and dwi are only part of the *Screening* session;
- Image volumes of BOLD fMRI data are provided as gzipped NIfTI 4D files, with an identifier corresponding to the following pattern:

sub-XX_ses-YY_task-ZZZ_acq-AA_bold.nii.gz, in which and XX and YY refer respectively to the subject and session id, ZZZ refers to the name of the task, and AA can be either ′PA′ or ′AP′ depending on the phase-encoding direction. Corresponding events files are available under the

sub-XX_ses-YY_task-ZZZ_acq-AA_event.tsv name as well as single band reference images

sub-XX_ses-YY_task-ZZZ_acq-AA_sbref.nii.gz.

Although BIDS does not yet provide support for data derivatives, i.e. preprocessed and post-processed data, a similar directory tree structure was still preserved for this content.

### Derived statistical maps

Furthermore, the unthresholded statistical maps, obtained from the contrasts of the aforementioned experimental conditions (see Section Experimental Paradigms for full description), have been released in the *NeuroVault* public repository^54^ with the id=2138 (https://neurovault.org/collections/2138/).

## Technical Validation

### Data quality

**Figure 1:**
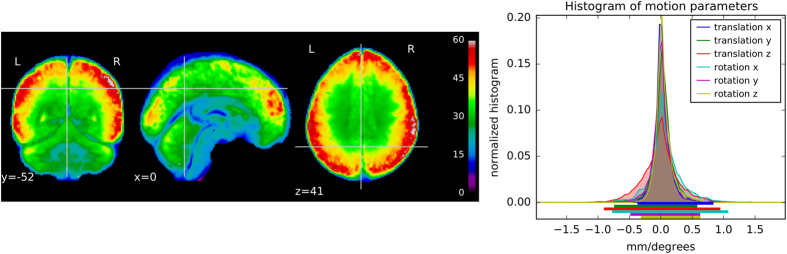
Global quality indices of the acquired data: tSNR map and motion magnitude distribution. (Left) The tSNR map displays the average of tSNR across all tasks and subjects. This shows values mostly between 30 and 60, with larger tSNR in cortical regions. (Right) Density of within-run motion parameters, pooled across subjects and tasks. Six distributions are plotted, for the six rigid-body parameter of head motion (translations and rotations are in mm and degrees, respectively). Each distribution is based on 73 k values, corresponding to all frame times for all acquisitions and subjects. Bold lines below indicate the 99% coverage of all distributions and show that motion parameters mostly remain confined to 1mm/1 degree across 99% of all acquired images.

In order to provide an approximate estimate of data quality, some standard measures are presented in Fig. 1 according to:

- The temporal SNR (tSNR), defined as the mean of each voxels′ time course divided by their standard deviation, on normalized and unsmoothed data averaged across all acquisitions. Its values are ~60 in the cortex. Given the high resolution of the data (1.5 mm isotropic), such values are indicative of a good image quality^55^;
- The histogram of the six rigid body motion estimates of the brain per scan, in mm/degree, together with their 99% coverage interval. One can notice that this interval ranges approximately within [−1,1]mm/degree, showing that excursions beyond 1 mm/degree motion are rare. No acquisition was discarded due to excessive motion (>2 mm/degree).

Other informal but systematic quality checks were performed using the PyPreprocess library (see Section Preprocessing).

### Relevance of the IBC dataset for brain mapping

Following the aforementioned procedures, one map per subject was obtained for each condition and each phase-encoding direction (PA/AP), with two exceptions: (1) some experiments were repeated between the Screening and ARCHI sessions, in which case only the latter was systematically considered; and (2) the RSVP Language task yields six maps, i.e. 3 in PA and 3 in AP phase-encoding direction respectively. There are, in overall, 59 conditions across tasks, which amounts to approximately 176 maps per subject, i.e. 2112 activation maps in total. Two high-level analyses of the activation maps, obtained upon processing and analysis of the individual data, are herein presented in order to assess whether they actually capture relevant cognitive networks.

#### Effect of subject identity and task stimuli on activation

**Figure 2:**
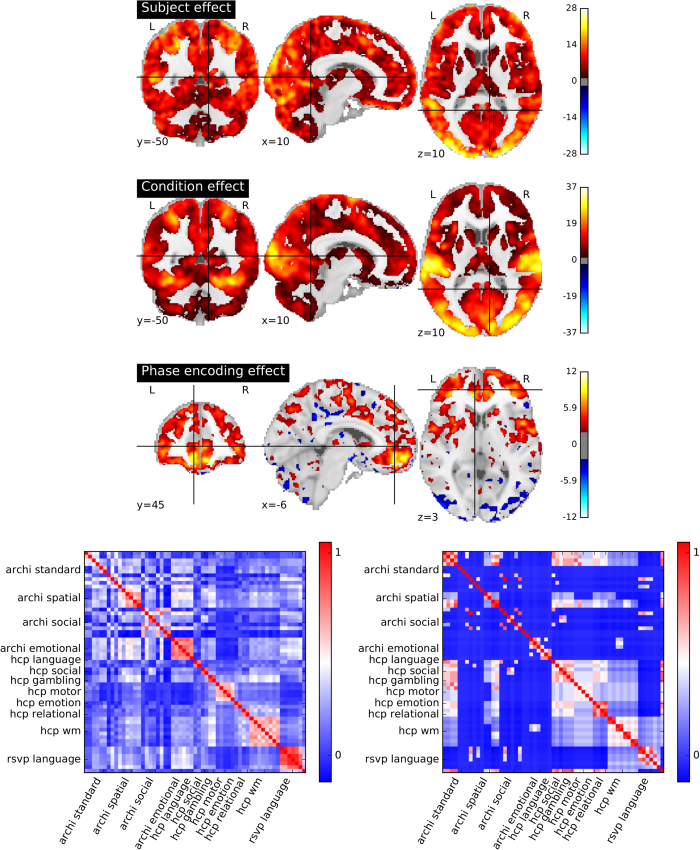
Overview of information conveyed by activation maps resulting from a first-level analysis. (top) Global effects of experimental subject condition, and phase-encoding direction. A per-voxel ANOVA breaks the variance of the set of brain maps into subject, experimental condition, and phase-encoding direction values. All maps are given in z-scale and thresholded at an FDR level of 0.05. (Bottom) Focusing on condition effect, the similarity between condition-related maps, averaged across subjects (left) is clearly related to the dissimilarity of the conditions, when these are characterized in terms of the Cognitive Atlas (right).

Taking into account the output of the GLM analysis for each acquisition, an assessment was performed at every voxel concerning how well the signal can be explained by (1) the effect of subject identity, (2) the condition, or (3) the phase-encoding direction. To assess the impact of these three factors, an *Analysis of Variance* (ANOVA) of all maps was computed and results from the first-level analysis of the data (hence 1688 maps, when discounting the duplicated experiments) were obtained for the aforementioned factors. The resulting statistical maps are displayed on the top of the Fig. 2. They show that both subject and condition effects are significant (uniformly significant at *p *< 0.05, *False Discovery Rate* (FDR)-corrected across voxels). Although condition effects are greater in sensory cortices, visual, auditory and somato-sensory regions in particular, subject effects are generally stronger. Phase-encoding direction effects are significant only in smaller regions, particularly at the frontal areas where distortions are known to have a stronger impact. Condition effects are consistently represented across participants, suggesting that the dataset fits the overall purpose of brain mapping at the individual level. Besides, separate analysis of different participants is also validated, because subjects effects are indeed non-negligible. Finally, the effect of phase-encoding direction cannot be dismissed, even after correction.

#### Similarity of brain activation patterns fits between-task similarity

Within-subject correlation matrices of all activation maps were computed as means to summarize the similarity between the response to experimental conditions. Note that they refer to a FFX analysis across replications (PA/AP phase-encoding and replications for the RSVP Language task). The average of the correlation matrices across subjects was then estimated in order to assess the pattern of similarity between tasks. Note that this does *not* involve averaging the fMRI maps across subjects. Besides, experimental conditions were also encoded according to the *Cognitive Atlas*' ontology (https://www.cognitiveatlas.org; see also^56^ for the link between each condition and cognitive labels). This labeling is described in detail in Table 4 (available online only). The correlation matrix of these cognitive descriptions was then computed, since such labeling offers an approximate characterization of the tasks. The two 59×59 correlation matrices (both map-wise and cognitive component-wise) are represented at the bottom of Fig. 2. They show clear similarities, together with discrepancies that are worthy of further investigation. Following the popular Representational Similarity Analysis approach^57^, the Spearman correlation was computed between the upper triangular coefficients, which amounted to 0.21 (*p *< 10^−17^; Pearson correlation: 0.34).

### Brain Coverage of Functional Activity

**Figure 3:**
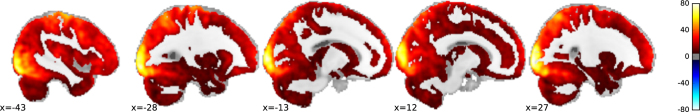
Group-level F-map, at a threshold of *p*<0.05 Bonferroni-corrected, representing the total area of the brain significantly covered by all tasks featuring the first release of the IBC dataset (FFX across tasks and subjects). One can readily see that all the brain is covered, with higher values in sensory cortices and weaker values for the temporal and pre-frontal cortex as well as subcortical structures.

A comprehensive brain coverage was already attained from the overall set of tasks comprising the first release of the IBC dataset (see Fig. 3). All brain areas are covered by this map.

Stronger effects are observed in medial sections of the occipital, parietal and frontal lobes as well as lateral areas of the occipital lobe plus posterior portions of both parietal and frontal lobes. By contrast, some temporal, pre-frontal and sub-cortical regions display weaker effects. Future releases may thus encompass tasks extensively addressing high-level visual object discrimination, including face selection, but also reward, decision-making and episodic memory. However, one must notice that brain coverage may not be fully attained due to MR-related technical restrictions. Indeed, some locations are particularly sensitive to e.g. coil sensitivity or intra-voxel dephasing, which can result in a reduced tSNR at specific functional brain regions.

## Usage Notes

The IBC project advocates the principles of data-sharing and reproducibility in neuroscience, as means to achieving transparency in research practice and consistency of results across time. Online access of data source and derivatives is assured by the Neuroinformatics Platform of the *Human Brain Project* as well as the *OpenfMRI* (Data Citation 1) and *NeuroVault* (consult Section Derived statistical maps) repositories.

This first release brings together a wide range of tasks covering many psychological domains. It features a total of 59 independent contrasts along with high brain coverage (see Section Brain Coverage of Functional Activity for further details). The collection of new data continues till year 2022 and more releases, e.g. especially dedicated to particular cognitive domains, are expected for the upcoming years. For instance, data from a set of tasks tackling in detail the visual system, such as visualization of naturalistic scenes or classic-retinotopy tasks, are expected in the upcoming releases. Moreover, tasks addressing higher cognitive functions pertaining to a finer coverage of prefrontal areas, like incentive salience or chronesthesia, are also included in the medium-term plan of the acquisitions. Ultimately, a comprehensive brain coverage of functional signatures linked to a large variety of cognitive functions is expected by the end of the project. In addition, future releases will also include high-resolution T1-, T2- and diffusion- weighted MRI images as well as T1- and T2- relaxometry data and myelin water fraction estimates.

The present release applies only BIDS Specification to data source. Nonetheless, a new version of BIDS providing support for preprocessed data is expected in the near future. The upcoming releases of the IBC dataset should comply with the normative organization of preprocessed data.

## Additional information

**How to cite this article**: Pinho, A. L. *et al*. Individual Brain Charting, a high-resolution fMRI dataset for cognitive mapping. *Sci. Data* 5:180105 doi: 10.1038/sdata.2018.105 (2018).

**Publisher’s note**: Springer Nature remains neutral with regard to jurisdictional claims in published maps and institutional affiliations.

## Supplementary Material



## Figures and Tables

**Table 1:** Demographic data of the participants.

Subject ID	Age	Sex	Handedness score
sub-01	39.5	M	0.3
sub-02	32.8	M	1
sub-04	26.9	M	0.8
sub-05	27.4	M	0.6
sub-06	33.1	M	0.7
sub-07	38.8	M	1
sub-08	36.5	F	1
sub-09	38.5	F	1
sub-11	35.8	M	1
sub-12	40.8	M	1
sub-13	28.2	M	0.6
sub-14	28.3	M	0.7
Age stands for the participants' age upon recruitment.			

**Table 2:** Plan of the MRI data acquisitions for the first five sessions.

Session	Modality	Task	Duration (min:sec)	Repetitions
Screening	T1-weighted	-	7:46	1
	T2-weighted	-	4:43	1
	T2 FLAIR	-	5:17	1
	DW-MRI	-	5:16	1
	2D Spin-Echo	-	0:31	PA(×2) + AP(×2)
	BOLD fMRI	ARCHI Spatial	5:46	PA + AP
	BOLD fMRI	ARCHI Standard	8:58	PA + AP
ARCHI	2D Spin-Echo	-	0:31	PA(×2) + AP(×2)
	BOLD fMRI	ARCHI Standard	5:46	PA + AP
	BOLD fMRI	ARCHI Spatial	8:58	PA + AP
	BOLD fMRI	ARCHI Social	9:18	PA + AP
	BOLD fMRI	ARCHI Emotional	7:54	PA + AP
HCP1	2D Spin-Echo	-	0:31	PA(×2) + AP(×2)
	BOLD fMRI	HCP Emotion	5:12	PA + AP
	BOLD fMRI	HCP Gambling	6:50	PA + AP
	BOLD fMRI	HCP Motor	6:44	PA + AP
	BOLD fMRI	HCP Language	8:12	PA + AP
HCP2	2D Spin-Echo	-	0:31	PA(×2) + AP(×2)
	BOLD fMRI	HCP Relational	10:56	PA + AP
	BOLD fMRI	HCP Social	7:06	PA + AP
	BOLD fMRI	HCP WM	10:40	PA + AP
RSVP Language	2D Spin-Echo	-	0:31	PA(×2) + AP(×2)
	BOLD fMRI	RSVP Language	10:54	PA(×3) + AP(×3)
This set of session constitutes the first release of the IBC dataset. A BOLD run refers to the acquisition of fMRI data on one single task. There were two BOLD runs, corresponding to PA- and AP- phase-encoding directions for each task during a session. As an exception, the session dedicated to the RSVP Language task included three runs for each phase-encoding direction. In addition, the experimental paradigm of this task displays different stimuli and has different onsets across repetitions. The 2D Spin-Echo AP/PA maps were always acquired before the runs dedicated to the collection of BOLD fMRI data and repeated afterwards.				

**Table 3:** Imaging parameters used for the acquisitions of the first IBC data release.

Modality	Voxel size (mm)	Slice orientation	Flip angle	TR (ms)	TE (ms)	FoV [x,y,z] (mm)	Acceleration	Other
T1-weighted MPRAGE	1 isotropic	sagittal	9°	2300	2.98	256,256,176	-	-
T2-weighted	0.9 isotropic	sagittal	-	3200	419	230,230,160	2 (GRAPPA)	-
T2 FLAIR	0.9 isotropic	sagittal	-	5000	396	230,230,160	3 (GRAPPA)	-
Diffusion	2 isotropic	axial	90°	9000	66	240,240,140	2 (GRAPPA)	B-value: 1500s.mm^−2^ Q=20 directions
fMRI	1.5 isotropic	axial	74°	2000	27	192,192,140	2 (GRAPPA)×3 (MB)	interleaved slice order
SE	1.5 isotropic	axial	74°	7680	46	192,192,140	2 (GRAPPA)	-
Note on the abbreviations that are not explicitly mentioned in the main text: TR = *Repetition Time*; TE = *Echo Time*; FoV = *Field of View*; and GRAPPA = *Generalized Autocalibrating Partially Parallel Acquisitions*.								

**Table 4:** Cognitive labels associated with the experimental conditions present across all IBC tasks.

Task	Condition	Cognitive Labels
ARCHI Standard	audio left button press	response selectionresponse executionleft finger response executionauditory sentence recognition
	audio right button press	response selectionresponse executionright finger response executionauditory sentence recognition
	video left button press	response selection response execution left finger response execution
	video right button press	response selection response execution right finger response execution
	horizontal checkerboard	horizontal checkerboard
	vertical checkerboard	vertical checkerboard
	audio sentence	auditory sentence recognition
	video sentence	visual word recognition sentence processing
	audio computation	auditory arithmetic processing
	video computation	visual arithmetic processing sentence processing
ARCHI Spatial	saccades	visual tracking
	rotation hand	response selection visual body recognition hand-chirality recognition
	rotation side	response selection visual body recognition hand side recognition
	object grasp	response selection response execution right finger response execution visual tool recognition grasping
	object orientation	response selection response execution right finger response execution visual tool recognition
ARCHI Social	mechanistic audio	auditory sentence recognition story comprehension
	mechanistic video	visual word recognition sentence processing
	triangle mental	animacy perception animacy decision motion detection
	triangle random	motion detection
	false belief audio	auditory sentence recognition story comprehension theory-of-mind
	false belief video	visual word recognition sentence processing theory-of-mind
	speech sound	voice perception
	non speech sound	sounds perception
ARCHI Emotional	face gender	visual face recognition gender discrimination
	face control	visual face recognition
	face trusty	visual face recognition facial trustworthiness recognition
	expression intention	emotion expression identification facial trustworthiness recognition
	expression gender expression control	emotion expression identification gender discrimination emotion expression identification
HCP Emotion	shape	visual form recognition feature comparison response selection response execution
	face	feature comparison response selection response execution emotional face recognition
HCP Gambling	punishment	response selection response execution punishment processing
	reward	response selection response execution reward processing
HCP Motor	left hand	response execution left finger response execution
	right hand	response execution right finger response execution
	left foot	response execution left toe response execution
	right foot	response execution right toe response execution
	tongue	response execution tongue response execution
HCP Language	story	response selection response execution auditory sentence recognition story comprehension
	math	response selection response execution auditory arithmetic processing
HCP Relational	relational	visual form recognition feature comparison response selection response execution relational comparison visual pattern recognition
	match	visual form recognition feature comparison response selection response execution visual pattern recognition
HCP Social	mental	response selection response execution animacy perception animacy decision motion detection
	random	response selection response execution motion detection
HCP Working Memory	0-back body	response execution working memory body maintenance visual body recognition
	2-back body	response execution working memory updating body maintenance visual body recognition
	0-back face	response execution working memory face maintenance visual face recognition
	2-back face	response execution working memory updating face maintenance visual face recognition
	0-back tools	response execution working memory visual tool recognition tool maintenance
	2-back tools	response execution working memory updating visual tool recognition tool maintenance
	0-back place	response execution working memory place maintenance visual place recognition
	2-back place	response execution working memory updating place maintenance visual place recognition
RSVP Language	complex	working memory visual word recognition word maintenance sentence processing syntactic parsing
	simple	working memoryvisual word recognition word maintenance sentence processing
	jabberwocky	working memory visual pseudo word recognition sentence processing
	word list	working memory visual word recognition word maintenance
	pseudoword list	working memory
		visual pseudo word recognition
	consonant string	working memory string maintenance visual string recognition
	probe	response selection response execution
The tags are obtained from the Cognitive Atlas: https://www.cognitiveatlas.org. Such labels provide an approximate description of the underlying cognitive components that are implied in the performance of the conditions.		
